# Differential Neuroprotective Effects of *N*-Acetylcysteine against Dithianon Toxicity in Glutamatergic, Dopaminergic, and GABAergic Neurons: Assessment Using Zebrafish

**DOI:** 10.3390/antiox12111920

**Published:** 2023-10-27

**Authors:** Amit Banik, Juneyong Eum, Byung Joon Hwang, Yun Kee

**Affiliations:** 1 Interdisciplinary Graduate Program in Environmental and Biomedical Convergence, College of Biomedical Science, Kangwon National University, Chuncheon 24341, Republic of Korea; natureamitbanik@kangwon.ac.kr (A.B.); eum1230@kangwon.ac.kr (J.E.); 2 Department of Molecular Bioscience, College of Biomedical Science, Kangwon National University, Chuncheon 24341, Republic of Korea; bjhwang@kangwon.ac.kr; 3 Division of Biomedical Convergence, College of Biomedical Science, Kangwon National University, Chuncheon 24341, Republic of Korea

**Keywords:** dithianon, developmental neurotoxicity, antioxidants, *N*-acetylcysteine, betaine, neuroprotection, GABAergic neurons, zebrafish

## Abstract

Despite the widespread agricultural use of dithianon as an antifungal agent, its neurotoxic implications for humans and wildlife have not been comprehensively explored. Using zebrafish embryonic development as our model, we found that dithianon treatment induced behavioral alterations in zebrafish larvae that appeared normal. Detailed quantitative analyses showed that dithianon at ≥0.0001 µgmL^−1^ induced cytoplasmic and mitochondrial antioxidant responses sequentially, followed by the disruption of mitochondrial and cellular homeostasis. Additionally, dithianon at 0.01 and 0.1 µgmL^−1^ downregulated the expressions of glutamatergic (*slc17a6b*), GABAergic (*gad1b*), and dopaminergic (*th*) neuronal markers. Contrarily, dithianon upregulated the expression of the oligodendrocyte marker (*olig2*) at concentrations of 0.001 and 0.01 µgmL^−1^, concurrently suppressing the gene expression of the glucose transporter *slc2a1a*/*glut1*. Particularly, dithianon-induced increase in reactive oxygen species (ROS) production was reduced by both *N*-acetylcysteine (NAC) and betaine; however, only NAC prevented dithianon-induced mortality of zebrafish embryos. Moreover, NAC specifically prevented dithianon-induced alterations in glutamatergic and dopaminergic neurons while leaving GABAergic neurons unaffected, demonstrating that the major neurotransmission systems in the central nervous system differentially respond to the protective effects. Our findings contribute to a better understanding of the neurotoxic potential of dithianon and to developing preventive strategies.

## 1. Introduction

The negative effects of chemical contaminants on the ecosystem and animal life have become a global concern. Despite the contribution of xenobiotics, such as pesticides and pharmaceutical compounds, to the growth of agricultural industries and the control of infectious diseases, they are harmful to the environment and endanger the health of humans and aquatic non-target organisms via surface or underground water contamination [1]. Identification and characterization of environmental chemicals with potential developmental neurotoxicity that result in cognitive and behavioral alterations and neurodegeneration remain challenging. 

Dithianon (5,10-dioxo-5,10-dihydronaphtho[2,3-b][1,4]dithiin), a quinone-based fungicide, has been widely used as a broad-range foliar antimycotic to treat various fungal infections in grains, fruits, and vegetables since the 1960s [2,3]. Dithianon binds non-specifically to the thiol group of cysteine residues in proteins, causing structural changes and enzymatic inhibition [4]. Although dithianon is considered safe in terms of acute toxicity [2], it acts as a pro-oxidant and suppresses mycelial growth and conidial germination in filamentous fungi [5]. Furthermore, it causes cell wall breakdown and reactive oxygen species (ROS) accumulation in yeast (*Saccharomyces cerevisiae*) [6]. Moreover, dithianon can cause immune modulation in human monocyte-derived macrophages [7], exert dopaminergic neurotoxicity in worms (*Caenorhabditis elegans*) [8], and reduce activities of succinate dehydrogenase and glycerol-3-phosphate dehydrogenase in the mitochondrial respiratory chain in the muscles of bumble bees (*Bombus terrestris*) [9]. However, the associated developmental neurotoxicity and modes of action of dithianon are poorly understood.

ROS act as cell signaling molecules in both normal and pathological biological processes. Mitochondria are among the most sophisticated responsive systems in the body. They are the principal generators of ROS, and failure in their function can induce oxidative stress and damage to other cellular compartments and molecular machinery, resulting in the progression of neurological diseases [10,11,12]. *N*-acetylcysteine (NAC) is an antioxidant, anti-inflammatory, and tumor-inhibiting agent [13,14] that reduces ROS production, lipid peroxidation, and apoptosis [15]. Betaine (*N*,*N*,*N*-trimethyl glycine) is an antioxidant that serves as an osmolyte to maintain cell volume, reverse stress-induced aberrant cellular methylation [16], reduce rotenone neurotoxicity, and improve mitochondrial respiration and oxidative stress [17,18,19].

Neurons communicate with each other through neurotransmitters. The three most important neurotransmitters in the CNS are glutamate, gamma-aminobutyric acid (GABA), and dopamine. Glutamate and GABA are the major excitatory and inhibitory neurotransmitters regulating neuronal excitability in the brain, and dopamine plays a role in the brain’s reward system and coordination of body movement. The intricate interplay among these neurotransmitters is crucial for the precise development and proper functioning of the brain. Disruption of neuromodulation has been linked to various neurological conditions, including depression, schizophrenia, Parkinson’s disease, and other neurodegenerative disorders [20,21]. In addition, glial cells support the growth and function of neurons, sustain the ionic environment of nerve cells, regulate the speed of nerve signal transmission, alter synaptic action by regulating neurotransmitter consumption, and assist in healing neurological damage [22,23]. A precise assessment of the developmental neurotoxicity of environmental chemicals is essential for understanding the disruption of neural circuits in the CNS and preventing neurological and psychiatric disorders.

Zebrafish (*Danio rerio*) is a useful vertebrate model in studies on hazard identification, particularly in toxicological research of environmental chemicals [24]. Tiny, transparent embryos of zebrafish can be obtained in large numbers, allowing the visualization of tissue development in live animals [25,26,27]. As most risk assessments of potential environmental factors are currently restricted to acute toxicity within limited concentration ranges, they may fail to identify unseen neurotoxicity resulting in alterations of neuronal functions in the brain, thus obscuring precise prevention strategies. To overcome this limitation, we investigated the developmental neurotoxicity of dithianon in a broad concentration range at the behavioral and molecular levels using zebrafish and the potential protective effects of the antioxidants NAC and betaine in preventing dithianon-induced developmental alterations. Our findings revealed the differential toxicity of dithianon and the potency of the protectants in embryonic development.

## 2. Materials and Methods

### 2.1. Zebrafish Maintenance

Wild-type AB zebrafish were obtained from the Zebrafish International Resource Center (Eugene, OR, USA). Adult zebrafish were raised in balanced saltwater at 27.5 °C under a 14/10 h light/dark cycle. Zebrafish eggs were obtained from 3–12-month-old adult fish by natural spawning and reared in E3 medium (5 mM NaCl, 0.33 mM MgSO_4_, 0.33 mM CaCl_2_, and 0.17 mM KCl) at 28.5 °C. The embryos were staged as hours post-fertilization (hpf) and days post-fertilization (dpf) according to Kimmel staging [28]. The procedures were approved by the Animal Use and Ethics Committee of Kangwon National University (KW-171227-2) and performed in accordance with the Institutional Animal Care and Use Committee principles and the National Research Council’s Guide for the Care and Use of Laboratory Animals.

### 2.2. Chemical Treatment

Dithianon (45462; Sigma-Aldrich Corp., St. Louis, MO, USA) was dissolved in dimethyl sulfoxide (DMSO) (D5879; Sigma-Aldrich Corp.) and diluted with E3 medium. Zebrafish embryos in the 50% epiboly stage (5 hpf) were treated with dithianon at 0, 0.0001, 0.001, 0.01, 0.1, 0.2, and 0.5 µgmL^−1^ concentrations in 5 mL of 0.5% DMSO/E3 medium in 35 mm Petri dishes and incubated at 28.5 °C; 25 zebrafish embryos in a Petri dish were used for each treatment, and the experiments were repeated at least three times. Developmental toxicity was examined at various time points: 28 hpf during early organogenesis, 50 hpf during hatching period, 3 dpf during early differentiation of the neurons involved in the major neurotransmission systems, and 6 dpf for larval behavior.

We tested the ability of NAC (A7250; Sigma-Aldrich Corp.) and betaine (B2629; Sigma-Aldrich Corp.) to alleviate dithianon-induced toxicity in zebrafish embryos using previously described methods [29,30,31]. Briefly, 25 zebrafish embryos in the 50% epiboly stage were treated with either 50 µM NAC, 50 mM betaine, or cotreated with 0.1 µgmL^−1^ dithianon. Treatments were administered in 5 mL of 0.5% DMSO/E3 medium in 35 mm Petri dishes, with 25 embryos per dish. Each experiment was repeated at least thrice.

### 2.3. Bright-Field Imaging and Survival Test

Morphological abnormalities in zebrafish embryos were monitored using bright-field imaging. Briefly, the embryos were anesthetized with 0.02% tricaine (Sigma-Aldrich Corp.) in E3 medium, mounted with 3% methylcellulose (Sigma-Aldrich Corp.), and bright-field images of the embryos were captured using a SZX16 fluorescent stereoscope (Olympus, Tokyo, Japan) equipped with an AxioCam camera (Carl Zeiss, Overkochen, Germany) and Zeiss Zen 3.4 (Blue edition) software (Carl Zeiss) [32].

The mortality of the embryos treated with dithianon from the 50% epiboly stage was examined until 4 dpf using the Olympus stereoscope. Survival rate of the 25 embryos treated with 0.1 µgmL^−1^ dithianon in the 50% epiboly stage was monitored at 5 dpf with or without co-treatment of NAC or betaine. All experiments were repeated at least three times.

### 2.4. Locomotive Behavior Analysis

Behavioral tests of zebrafish larvae treated with dithianon were performed in the 6 dpf stage [32]. Before the testing day, each larva was transferred to an individual well of a 24-well plate with 2 mL of E3 medium and incubated at 28.5 °C for 16 h. The 24-well plate was placed in a Danio Vision Observation Chamber (Noldus Information Technology, Wageningen, The Netherlands), and a light–dark transition test was conducted using a high-quality video tracking system with a 5 min acclimation period in the dark and six alternating cycles of 5 min intervals of light and dark stimuli. Larval locomotor activity was tracked using EthoVision XT 14 software (Noldus). Data points were exported to Microsoft Excel to generate graphs.

### 2.5. Quantitative Reverse Transcription Polymerase Chain Reaction (qRT-PCR)

Twenty-five embryos from each exposure group were collected in a 1.5 mL tube at the 28- or 72 hpf stage, and then 500 µL of TRI Reagent™ solution was added (AM9738; Thermo Fisher Scientific, Waltham, MA, USA). Thereafter, 100 µL of chloroform was added to separate the organic and inorganic phases, followed by 250 µL of isopropanol to precipitate the RNA. The total RNA was extracted using the Qiagen RNeasy Mini Kit (74104, 40724; Qiagen, Hilden, Germany), and RNA concentration was measured using a Nanodrop Lite Spectrophotometer (Thermo Fisher Scientific). cDNA was synthesized using Superscript III Reverse Transcriptase (18080093; Thermo Fisher Scientific) according to the manufacturer’s instructions. Quantitative PCR (qPCR) was performed on the QuantStudio 1 Real-Time PCR system (Thermo Fisher Scientific) using SYBR Green with low ROX (RT500M; TOPreal™ SYBR Green qPCR PreMIX; Enzynomics, Daejeon, Republic of Korea); b-actin was used as the control. Each sample was run in triplicate. Gene expression was normalized to the expression level of *b-actin* (*actb1*). The transcripts were quantified using the 2^−ΔΔCt^ method. The qPCR primers are listed in the Supplementary file (Table S1).

### 2.6. Reactive Oxygen Species Detection

The control and treated embryos were dechorionated at 50 hpf, placed in a small Petri dish (eight embryos per group) with 5 mL of 1 µgmL^−1^ 2′,7′-dichlorofluorescein diacetate (35845-1G; Sigma-Aldrich, Burlington, MA, USA) in E3 medium with 0.1% DMSO, and incubated in the dark at 23 °C for 30 min. The embryos were anesthetized with 0.02% tricaine, mounted with 3% methylcellulose on microscope slides, and imaged using a SZX16 fluorescence microscope (Olympus). Fluorescence intensity in the region of interest of each sample was measured using ImageJ 1.53 software (http://rsbweb.nih.gov/ij/, accessed on 1 May 2021).

### 2.7. Statistical Analysis

All statistical analyses were performed, and graphs were created using GraphPad Prism (v.9; GraphPad Software, San Diego, CA, USA). The data are presented as quantitative values, which are expressed as mean ± standard deviation. To ensure statistical robustness, each experiment had a minimum of three independent tests. Differences between control and treatment groups were assessed after checking for homogeneity of variances with Levene’s test using a one-way analysis of variance (ANOVA) followed by Bonferroni post hoc multiple comparisons. All statistical analyses were carried out using SPSS version 24. To determine statistical significance, the levels of significance were set as follows: * *p* < 0.05, ** *p* < 0.01, and *** *p* < 0.001.

## 3. Results

### 3.1. Effects of Dithianon Exposure on Zebrafish Larval Development

**Figure 1:**
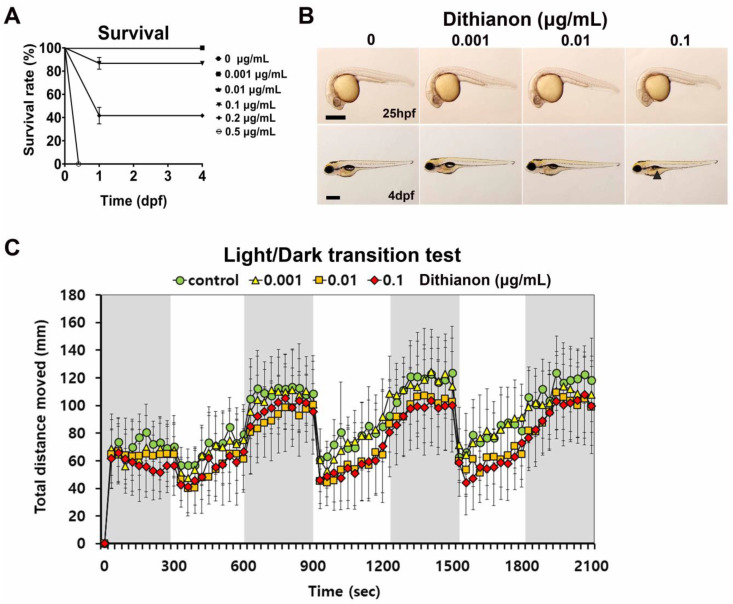
Anatomical and behavioral phenotyping reveals dithianon-induced toxicity during zebrafish larval development. (**A**) Survival rates of zebrafish embryos exposed to dithianon at concentrations of 0, 0.001, 0.01, and 0.1 µgmL^−1^ from 5 h post-fertilization (hpf) until 4 days post-fertilization (dpf). *n* = 5. (**B**) Lateral views of embryos subjected to bright field imaging at 25 hpf and 4 dpf. Black arrowhead, shrunken swim bladder; scale bar = 500 µm. n = 4. (**C**) Locomotive activity of 6 dpf larvae treated with dithianon as shown by the graphical representation of the average distance traveled every 30 s in the dark and light transitions. The data are provided as the means ± SD (*n* = 6).

To investigate the toxicity of dithianon during zebrafish embryonic development, the survival rate of zebrafish embryos exposed to 0.001–0.5 µgmL^−1^ dithianon was examined from 5 hpf to 4 dpf (Figure 1A). The survival rate of embryos exposed to 0.001 and 0.01 µgmL^−1^ dithianon was 100%. However, the mortality rate of embryos exposed to 0.1 µgmL^−1^ dithianon at 24 hpf was 18% ± 14%, and the remaining embryos survived through 4 dpf. The mortality rate of embryos exposed to 0.5 µgmL^−1^ dithianon was 100% at 12 hpf (Figure 1A). The median lethal dose of dithianon at 24 hpf was 0.141 µgmL^−1^. The morphological phenotypes of the dithianon-treated embryos were observed at 25 hpf and 4 dpf (Figure 1B). The embryos exposed to 0.1 µgmL^−1^ dithianon showed abnormalities such as a short body (28% ± 12%) at 25 hpf, and small eyes (43% ± 5%) and shrunken swim bladders (60% ± 5%) at 4 dpf (Figure 1B). However, the light–dark transition test showed reduced locomotive activity in 6 dpf larvae exposed to 0.01 µgmL^−1^ dithianon, although the larvae exhibited normal morphology (Figure 1C). These results suggest that behavioral assessment is an effective tool for detecting aberrant neurotoxicity in zebrafish larvae exposed to environmental chemicals.

### 3.2. Effects of Dithianon Exposure on Antioxidant Response and Cellular Homeostasis

**Figure 2:**
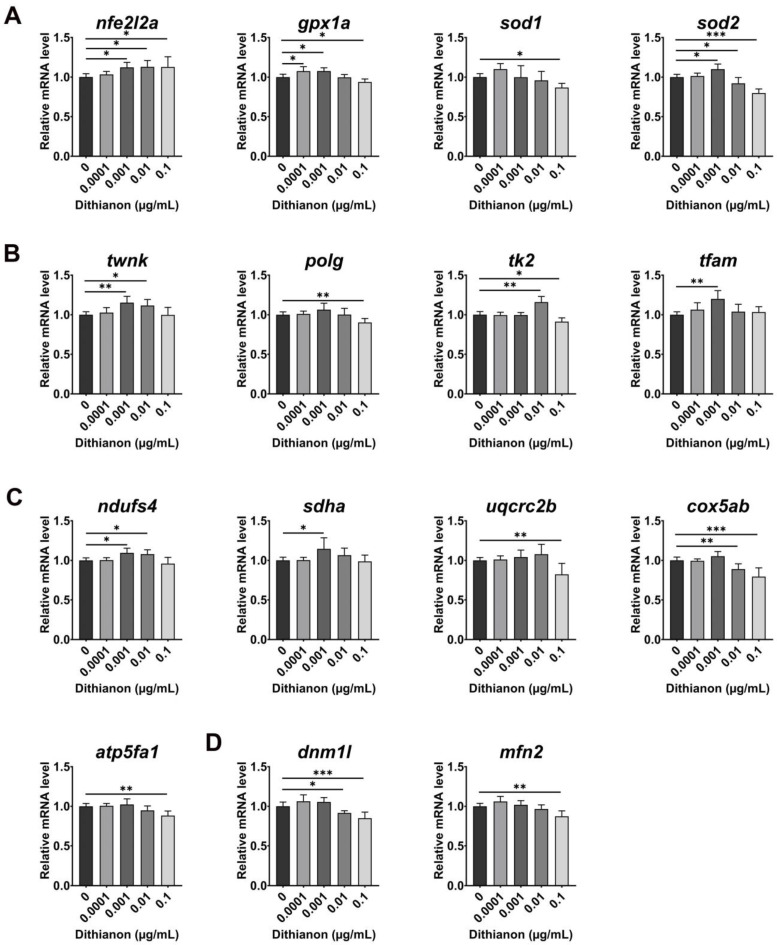
Dithianon alters antioxidant response and mitochondrial functions. (**A**) mRNA expression analysis of antioxidant response genes at 28 hpf using qRT-PCR: *nfe2l2a*, *gpx1a*, *sod1*, and *sod2*. (**B**) mRNA expressions of genes involved in mitochondrial DNA replication and transcription at 28 hpf: *twnk*, *polg*, *tk2*, and *tfam*. (**C**) mRNA expressions of mitochondrial respiratory complex genes at 28 hpf: *ndufs4* of complex I, *sdha* of complex II, *uqcrc2b* of complex III, *cox5ab* of complex IV, and *atp5fa1* of complex V. (**D**) mRNA expressions of mitochondrial fission and fusion markers at 28hpf: *dnm1l* and *mfn2*. The data are provided as the means ± SD (*n* = 4). * p < 0.05, ** p < 0.01, and *** p < 0.001.

We investigated the molecular basis of the dithianon-induced oxidative stress response in zebrafish using qRT-PCR. Dithianon exposure caused a dose-dependent decrease in the mRNA expression of *nfe2l2a* (nuclear factor erythroid-derived 2-like 2, also known as Nrf2), an upstream regulator in antioxidant genes (Figure 2A). Dithianon exposure upregulated the gene expressions of cytoplasmic and mitochondrial antioxidant enzymes *gpx1a* (glutathione peroxidase 1a) and *sod1* (superoxide dismutase 1) at 0.0001 µgmL^−1^ and that of *sod2* (mitochondrial superoxide dismutase 2) at 0.001 µgmL^−1^. Moreover, the expressions of *gpx1a*, *sod1*, and *sod2* were downregulated in embryos exposed to dithianon at 0.1 µgmL^−1^ (Figure 2A). These results suggest that dithianon-induced oxidative stress sequentially induces the expressions of cytoplasmic antioxidant genes and mitochondrial genes to maintain cellular homeostasis as an adaptive response to dithianon exposure at low doses. However, dithianon at high doses disrupted the antioxidant response.

We monitored mitochondrial mRNA expression during dithianon-induced oxidative response, including genes involved in the regulation of mitochondrial DNA (mtDNA) content and metabolism (Figure 2B). Exposure to 0.001 µgmL^−1^ dithianon upregulated the expressions of *twnk* (mtDNA helicase twinkle mtDNA helicase) and *polg* (mtDNA polymerase (DNA directed) gamma), which are essential for mtDNA replication. Furthermore, the expression of *tk2* (deoxyribonucleoside kinase thymidine kinase 2) for mtDNA synthesis considerably increased under 0.01 µgmL^−1^ dithianon treatment. Exposure to 0.001 µgmL^−1^ dithianon significantly upregulated the expression of *tfam* (mitochondrial transcription factor A, mitochondrial) (*p* < 0.01), which is essential for mtDNA transcription. On the contrary, the expressions of both *polg* and *tk2* were significantly downregulated following 0.1 µgmL^−1^ dithianon treatment (*p* < 0.01 and *p* < 0.05, respectively) (Figure 2B).

As the above results indicated that dithianon induces alterations in mtDNA replication and transcription, we examined the expression of components of mitochondrial respiratory complexes (Figure 2C). Dithianon exposure upregulated the expressions of *ndufs4* (NADH–ubiquinone oxidoreductase iron–sulfur protein 4 subunit) of complex I, *sdha* (succinate dehydrogenase A) of complex II, *uqcrc2b* (ubiquinol–cytochrome c reductase core protein II) of complex III at 0.001 and 0.01 µgmL^−1^, and that of *cox5ab* (cytochrome c oxidase subunit 5ab) of complex IV at 0.001 µgmL^−1^ compared with those in the untreated controls. On the contrary, the expressions of *uqcrc2b*, *cox5ab*, and *atp5fa1* (ATP synthase mitochondrial F1 complex alpha subunit) of complex V were significantly downregulated under 0.1 µgmL^−1^ treatment (*p* < 0.01, *p* < 0.01 and *p* < 0.001, respectively) compared with those in the untreated controls (Figure 2C). We also examined the expression of genes involved in the mitochondrial fission–fusion cycle. The expressions of *dnm1l* (mitochondrial fission marker dynamin 1-like) and *mfn2* (fusion marker mitofusin 2) were slightly upregulated in embryos exposed to 0.0001 µgmL^−1^ dithianon but dose-dependently downregulated in embryos exposed to 0.01 and 0.1 µgmL^−1^ (Figure 2D). These findings imply that dithianon triggers an antioxidant response at low doses but dysregulates it at high doses.

**Figure 3:**
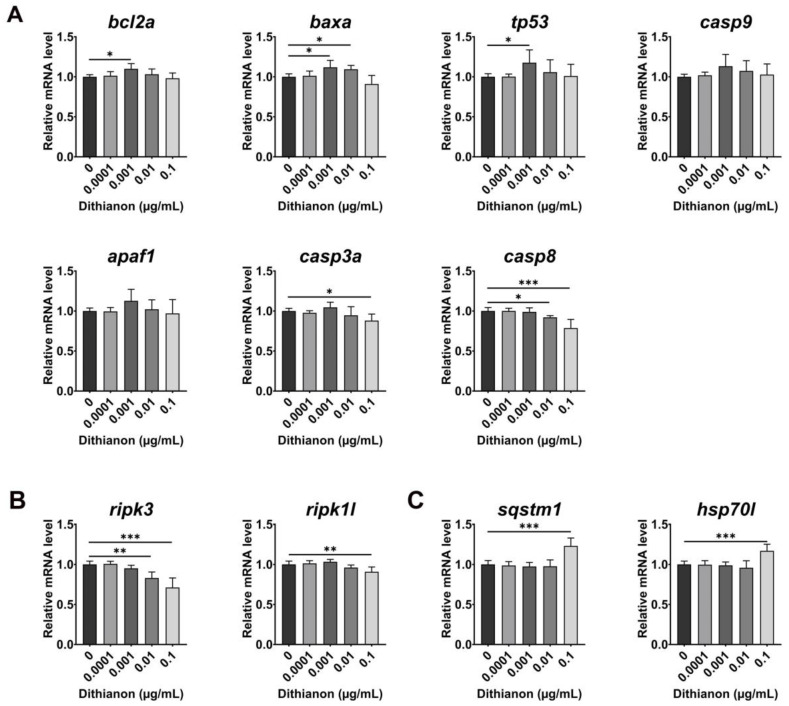
Dithianon triggers mitochondrial apoptotic pathway depending on its concentration. (**A**) mRNA expression analysis of the apoptotic genes in 28 hpf embryos treated with dithianon using qRT-PCR: *bcl2a*, *baxa*, *tp53*, *casp9*, *apaf1*, *casp3a*, and *casp8*. (**B**) mRNA expression of the necroptotic genes: *ripk3* and *ripk1l*. (**C**) mRNA expression of the autophagy adaptor *sqstm1* and molecular chaperon *hsp70l*. The data are provided as the means ± SD (*n* = 4). * p < 0.05, ** p < 0.01, and *** p < 0.001.

We examined the effect of dithianon on cell death (Figure 3A). The expression of mitochondrial apoptotic genes was upregulated in embryos exposed to 0.001 µgmL^−1^ dithianon at 28 hpf, namely, the anti-apoptotic gene *bcl2a* (BCL2 apoptosis regulator a), pro-apoptotic gene *baxa* (BCL2 associated X apoptosis regulator a), *tp53* (tumor protein p53), pro-apoptotic gene *casp9* (apoptosis-related cysteine peptidase 9), and activator of mitochondrial apoptotic signaling *apaf1* (apoptotic peptidase activating factor 1) compared with those in the untreated controls. Furthermore, the expression of *casp3a* (apoptosis-related cysteine peptidase a) was slightly upregulated under 0.001 µgmL^−1^ dithianon treatment, and those of both *casp3a* and *casp8* (apoptosis-related cysteine peptidase) were significantly downregulated under 0.1 µgmL^−1^ treatment (*p* < 0.001), compared with those in the untreated controls (Figure 3A). The expression of the necroptotic marker *ripk3* (receptor-interacting serine–threonine kinase 3) was significantly downregulated under 0.01 and 0.1 µgmL^−1^ dithianon treatments (*p* < 0.01 and *p* < 0.001, respectively), and that of *ripk1l* (receptor (TNFRSF)-interacting serine–threonine kinase 1, like) was also significantly downregulated under 0.1 µgmL^−1^ treatment (*p* < 0.01), compared with those in the untreated controls (Figure 3B). In particular, the expressions of the autophagy adaptor marker *sqstm1* (sequestosome 1) and stress-induced molecular chaperone *hsp70l* (heat shock cognate 70 kd protein, like) significantly increased in embryos exposed to 0.1 µgmL^−1^ dithianon (*p* < 0.001) compared with those in the untreated controls (Figure 3C). These results suggest that dithianon triggers mitochondrial apoptotic pathways and possibly the autophagy clearance system depending on its concentration.

### 3.3. Effects of Dithianon Exposure on Neural and Glial Development

**Figure 4:**
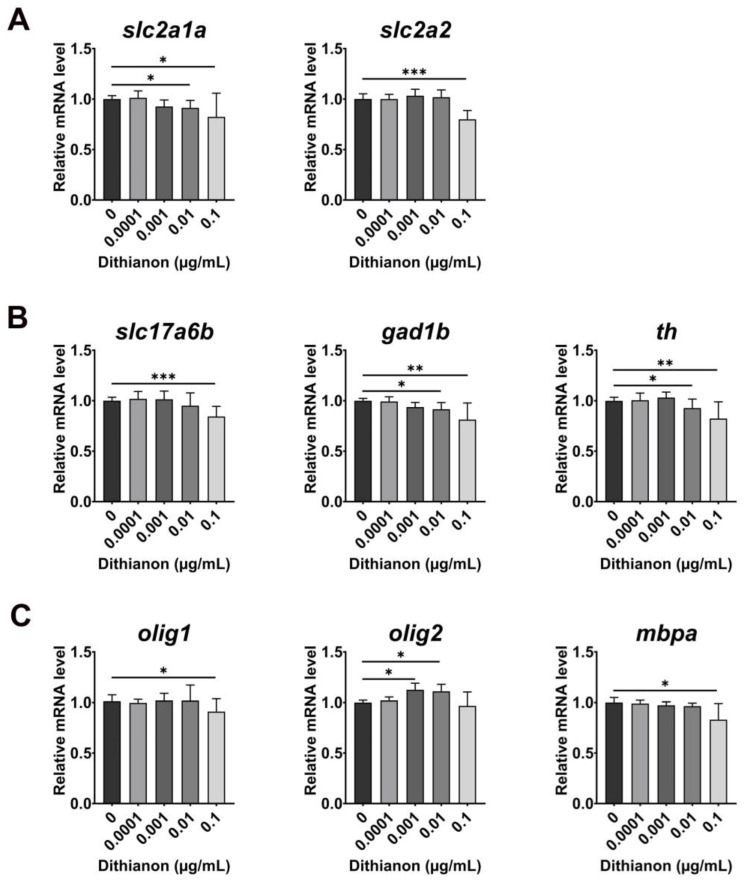
Dithianon specifically alters expression of genes essential for glucose transport, neurotransmission, and glial development in the central nervous system depending on its concentration. (**A**) mRNA expression analysis of glucose transporters, *slc2a1a* and *slc2a2*, at 3 dpf using qRT-PCR. (**B**) mRNA expressions of the glutamatergic excitatory neuronal marker *slc17a6b*, the GABAergic inhibitory neuronal marker *gad1b*, and the dopaminergic neuronal marker *th*, all at 3 dpf. (**C**) mRNA expressions of oligodendrocyte markers, *olig1* and *olig2*, as well as the myelination marker *mbpa*, at 3 dpf. The data are provided as the means ± SD (*n* = 4). * p < 0.05, ** p < 0.01, and *** p < 0.001.

We investigated whether dithianon-induced oxidative stress impairs glucose metabolism [33] by measuring the mRNA expression of glucose transporters in 3 dpf larvae exposed to dithianon (Figure 4A). Exposure of embryos to 0.01 µgmL^−1^ dithianon significantly reduced the level of *slc2a1a* (solute carrier family 2 member 1a)/*glut1* (*p* < 0.05), which is expressed in the cranial vasculature, endothelial cells, otic vesicles, and liver, compared with that in the untreated controls. In contrast, the level of *slc2a2* (solute carrier family 2 member 2)/*glut2*, a glucose sensor that is expressed in the brain, muscle, and liver, was unaffected in embryos treated with 0.01 µgmL^−1^ dithianon and significantly decreased with 0.1 µgmL^−1^ (*p* < 0.001) compared with that in the untreated controls (Figure 4A). These findings suggest that dithianon may disrupt glucose homeostasis regulated with *slc2a1a* and *slc2a2*, with the extent of disruption depending on the dithianon concentration.

Deficiencies in *slc2a1a* and *slc2a2* cause neurological disorders and aberrant neurotransmitter release [34,35]. Therefore, we examined the effects of dithianon on major neurotransmission systems in zebrafish embryonic development based on our result of dithianon-induced decrease in the expressions of *slc2a1a* and *slc2a2*. In embryos exposed to 0.01 and 0.1 µgmL^−1^ dithianon, the expression of *slc17a6b* (solute carrier family 17 member 6b), a glutamatergic excitatory neuronal marker, *gad1b* (glutamate decarboxylase 1b), a GABAergic inhibitory neuronal marker, and *th* (tyrosine hydroxylase), a dopaminergic neuronal marker, were downregulated in a dose-dependent manner (Figure 4B). Interestingly, the expression of *olig2* (oligodendrocyte lineage transcription factor 2), a marker of oligodendrocyte progenitor, was considerably upregulated in the embryos exposed to 0.001 and 0.01 µgmL^−1^ dithianon, whereas expressions of *olig1* (oligodendrocyte transcription factor 1), a marker of oligodendrocyte progenitor, and *mbpa* (myelin basic protein a), a myelination marker, were not markedly altered compared with those in the untreated controls (Figure 4C). The expressions of *olig1* and *mbpa* were significantly downregulated in embryos exposed to 0.1 µgmL^−1^ dithianon (*p* < 0.05) compared with those in the untreated controls. These results suggest that dithianon reduces the populations of glutamatergic excitatory, GABAergic inhibitory, and dopaminergic neurons but increases the oligodendrocyte progenitors in the developing central nervous system at 0.01 µgmL^−1^.

### 3.4. Protection Potency of N-Acetylcysteine and Betaine on Dithianon-Induced Toxicity

**Figure 5:**
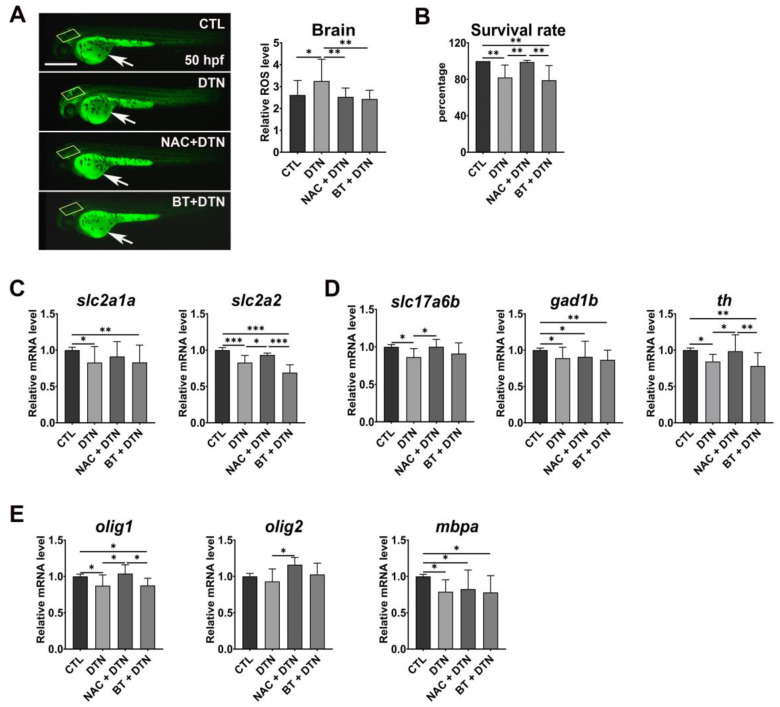
*N*-acetylcysteine and betaine differentially alleviate dithianon-induced developmental neurotoxicity. (**A**) Lateral views of 50 hpf whole embryos treated with 0.1 µgmL^−1^ dithianon and the antioxidants, NAC and betaine, and subjected to ROS detection assay using 2′,7′-dichlorofluorescein diacetate. Boxes, brain area to quantitate ROS level in the graph; arrows, nonspecific fluorescence in the yolk; scale bar = 500 µm. The graph shows the relative levels of ROS in the brain. The data are provided as the means ± SD (*n* = 4). (**B**) Survival rate of 5 dpf embryos co-treated with 0.1 µgmL^−1^ dithianon and NAC or betaine. The data are provided as the means ± SD (*n* = 5). (**C**) The mRNA expression analysis of the glucose transporters *slc2a1a* and *slc2a2* in 3 dpf larvae treated with 0.1 µgmL^−1^ dithianon and NAC or betaine using qRT-PCR. (**D**) mRNA expressions of neuronal genes *slc17a6b*, *gad1b*, and *th* in 3 dpf larvae. (**E**) mRNA expressions of glial genes *olig1*, *olig2*, and *mbpa* in 3 dpf larvae. The data are presented as the means ± SD (*n* = 6). * p < 0.05, ** p < 0.01, *** p < 0.001. CTL, control; DTN, dithianon; NAC, *N*-acetylcysteine; BT, betaine.

Consistent with the molecular disruption of the antioxidant pathway in embryos treated with 0.1 µg/mL ^−1^ dithianon (Figure 2A), ROS production increased in the larval brains of the dithianon-exposed zebrafish at 50 hpf compared with those in the untreated controls (Figure 5A). We then examined whether the antioxidants could alleviate the dithianon-induced alterations and found that both NAC and betaine significantly reduced the ROS levels in larvae treated with 0.1 µgmL^−1^ dithianon (*p* < 0.01) (Figure 5A). However, only NAC markedly prevented mortality in the larvae treated with 0.1 µgmL^−1^ dithianon (Figure 5B) and the dithianon-induced decrease in the expression of glucose transporter *slc2a2* but not that of *slc2a1a* (Figure 5C). These results indicate that NAC and betaine differentially ameliorated dithianon-induced developmental alterations.

In particular, the expression of *slc17a6b* in glutamatergic excitatory neurons and *th* in dopaminergic neurons in the central nervous system of larvae treated with 0.1 µgmL^−1^ dithianon was only significantly restored with NAC (*p* < 0.05); however, both antioxidants failed to restore the expression of *gad1b* in GABAergic inhibitory neurons (Figure 5D). NAC upregulated the expressions of the glial markers *olig1* (*p* < 0.05) and *olig2* (*p* < 0.05) but not that of *mbpa* in the larvae treated with 0.1 µgmL^−1^ dithianon (Figure 5E). These results suggest that NAC may be more effective than betaine in alleviating dithianon neurotoxicity in selective neuronal and glial cell populations, thereby reducing larval mortality.

Moreover, the supplementation of NAC or betaine did not affect the dithianon-induced decreases in expressions of *slc2a1a*, *gad1b*, and *mbpa* (Figure 5), although the individual treatment of embryos with 50 mM NAC alone markedly reduced mRNA expressions of *slc2a1a*, *gad1b*, and *mbpa*, among which the 50 mM betaine alone also reduced expressions of *gad1b* and *mbpa* (Figure S1).

## 4. Discussion

Dithianon is used worldwide as a fungicide in various agricultural products; however, its neurodevelopmental toxicity is poorly understood. Here, we investigated the developmental neurotoxicity of dithianon at the behavioral and molecular levels in zebrafish and examined the potential of antioxidants NAC and betaine in preventing dithianon-induced developmental toxicity.

The limited period of mortality of zebrafish embryos observed in this study may reflect the half-life of dithianon. Interestingly, our behavioral assessment revealed reduced locomotive activity of 6 dpf larvae treated with 0.01 µgmL^−1^ dithianon, although there were no noticeable anatomical abnormalities. Dithianon at concentrations ranging from 100 to 1000 µgmL^−1^ induced dopaminergic neuronal toxicity in live *C. elegans* worms [8], a concentration range at which zebrafish embryos cannot survive, indicating that *C. elegans* may be less susceptible to dithianon at concentrations used here, probably due to the reduced absorbance of the chemical through the cell wall. Dithianon toxicity was also examined in human monocyte cells at 0.029633, 0.29633, and 2.9633 µgmL-1 [7] and in yeast at 0.29633, 0.59266, and 1.1853 µgmL^−1^ [6].

Our study showed that dithianon has multifaceted molecular functions in zebrafish embryogenesis depending on its concentration. In the behavioral assessment, dithianon reduced locomotor activity in zebrafish larvae even when anatomical abnormalities were not detected. Additionally, molecular assessment of zebrafish larvae revealed dithianon-induced disruption of mitochondrial and glucose homeostasis, which may underlie the toxic effect of dithianon. Both NAC and betaine prevented dithianon-induced ROS production, but only thiol-based NAC reduced embryonic mortality and neurotoxicity of selective neurotransmission systems; however, none of them restored or worsened the expressions of *slc2a1a* glucose transporter, *gad1b* in the GABAergic inhibitory neurons, and *mbpa* in myelinating glia in the larvae treated with 0.1 µgmL^−1^ dithianon. Our findings indicate that dithianon differentially affects the major neurotransmission systems in the CNS and that NAC could be more effective than betaine in reducing dithianon-induced toxicity.

In the present study, qRT-PCR analysis showed that dithianon significantly induced the antioxidant response in zebrafish, as indicated by the dose-dependent upregulation of *nfe2l2a*. However, its downstream genes showed a biphasic response to dithianon: The expressions of the antioxidant enzyme-coding genes *gpx1a*, *sod1*, and *sod2* were upregulated by dithianon at low concentrations, but their expressions were downregulated by dithianon at high concentrations even when *nfe2l2a* expression was upregulated (Figure 2). Although previous studies have shown dithianon toxicity within limited concentration ranges that cause monotonous responses in the effectors in each cell or model organism [6,7,8,9], our wider-range profiling allowed us to reveal the biphasic response of the effector genes. These results also suggest that dithianon triggers the *nfe2l2a*-driven antioxidant response sequentially from the cytoplasm to the mitochondria in embryos treated at low concentrations but disrupts it at high concentrations.

Furthermore, dithianon exposure concentration-dependently interrupted mitochondrial homeostasis. A previous study showed that dithianon at 100–1000 µgmL^−1^ induced mitochondrial fragmentation in worms [8]. Although we did not observe mitochondrial fragmentation at lower concentrations at which zebrafish larvae survived, we detected molecular alterations indicating functional interruptions. In addition, the glucose transmitter-encoding gene *slc2a1a* (*glut 1*) is required for the formation of the blood–brain barrier [36], and *slc2a2* (*glut2*) is important for glucose sensing in neural circuits of the central nervous system [37]. Our results suggest that dithianon-induced downregulation of the glucose transporters may cause an aberrant level of neurotransmissions resulting in interrupted communication among neurons.

Moreover, in this study, the expression of apoptotic markers was upregulated by dithianon at low concentrations; however, there was no change in the ratio of *bcl-2a* to *baxa*, a predictor of apoptotic susceptibility, indicating dithianon-induced apoptotic pressure without cell death. In contrast, the expression of most apoptotic and necroptotic markers was downregulated by dithianon at high concentrations. The expression of the molecular chaperone *hsp70l* and autophagy adaptor *sqstm1* was considerably upregulated, which could have protected zebrafish against dithianon-induced cellular toxicity [8].

In this study, we demonstrated dithianon-induced toxicity in the three major neurotransmission systems of the central nervous system, whereas it was recently shown that dithianon caused only dopaminergic neuronal toxicity in *C. elegans* worms at very high concentrations [8]. In contrast to the neuronal response observed in our study, there was an increase in the population of *olig2* oligodendrocyte progenitors in the larvae treated with 0.001 and 0.01 µgmL^−1^ dithianon, suggesting that the glial cells that support neuronal populations may compensate for dithianon-induced neuronal loss. Nonetheless, dithianon-induced developmental alterations in neurotransmission systems may disrupt neural connections and communication, possibly resulting in neurological and psychiatric disorders.

Both antioxidants NAC and betaine reduced dithianon-driven ROS production in the brain; however, early embryonic mortality was reduced only with NAC, suggesting that ROS generation is not the direct cause of embryonic death under 0.1 µgmL^−1^ dithianon treatment. Dithianon-induced decrease in glutamatergic and dopaminergic neuronal populations and *olig1* and *olig2* glial cell populations was also significantly restored with NAC but not with betaine. However, the populations of GABAergic neurons and *mbpa* myelinated glial cells were not restored with NAC or betaine under similar conditions. These findings indicate that the neuronal populations of different neurotransmission systems respond differentially to dithianon-induced oxidative stress and neurotoxicity and that neurotoxicity is also differentially alleviated by NAC and betaine. Dithianon is known to react with thiol groups in peptides, including glutathione, which modulates the physiological levels of ROS and the cellular oxidative stress response [38]. Accordingly, we speculate a possible mechanism underlying the distinct protective effect of NAC against dithianon-induced neurotoxicity: The thiol-based antioxidant NAC may scavenge dithianon-induced thiyl radicals, spare the antioxidant glutathione from dithianon attack, and directly attack the carbonyl group of dithianon, inactivating its function.

Developmental neurotoxicity from dithianon is poorly understood, particularly in vertebrates. To this end, dithianon was recently shown to induce dopaminergic neurotoxicity in worms, and NAC alleviated this effect [8]. In the present study, we demonstrated that dithianon at lower concentrations causes developmental neurotoxicity in the three major neurotransmission systems and that antioxidants could serve as potential protectants, using zebrafish as a vertebrate model. It is not yet clear why altered GABAergic neurons were less responsive to the protectants than glutamatergic and dopaminergic neurons. Our findings provide a basis for further studies on the differential potency of protectants against the toxicity of dithianon and similar environmental chemicals.

## 5. Conclusions

Our findings distinguished the differential toxicity of dithianon at different concentrations and the potency of the protectants NAC and betaine in embryonic development using zebrafish. In particular, behavioral and molecular assessments demonstrated that dithianon exposure altered the major neurotransmission systems in zebrafish larvae. Moreover, the altered GABAergic neurons were less responsive to NAC than glutamatergic and dopaminergic neurons, indicating the differential potency of protectants against the toxicity of environmental chemicals. Developing neuronal populations interact with each other to generate neuronal circuits with proper connections and functions in the central nervous system. Therefore, it is essential to develop a strategy for the integrative assessment of chemical toxicity and protection at the organismal level.

## Data Availability

Data are contained within the article or Supplementary Material.

## Supplementary Materials

The following supporting information can be downloaded at: https://www.mdpi.com/article/10.3390/antiox12111920/s1, Table S1: List of primers used in qRT-PCR; Figure S1: Effects of *N*-acetylcysteine and betaine in 3 dpf zebrafish larvae. (A) The mRNA expression analysis of the glucose transporters *slc2a1a* and *slc2a2* in 3 dpf larvae treated with 50 µM *N*-acetylcysteine (NAC) or 50 mM betaine (BT) using qRT-PCR. (B) mRNA expressions of neuronal genes *slc17a6b*, *gad1b*, and *th*, all of which are involved in major neurotransmission systems. (C) mRNA expressions of glial genes, *olig1*, *olig2*, and *mbpa*. The mRNA expression data are provided as the means ± SD (*n* = 3). * *p* < 0.05, ** *p* < 0.01, *** *p* < 0.001. CTL, control; NAC, *N*-acetylcysteine; BT, betaine.
