# Supplementary material for: Differential Neuroprotective Effects of N-Acetylcysteine against Dithianon Toxicity in Glutamatergic, Dopaminergic, and GABAergic Neurons: Assessment Using Zebrafish
Source: Antioxidants (Basel). 2023 Oct 27;12(11):1920. doi: 10.3390/antiox12111920 (PMC10668936; doi:10.3390/antiox12111920)
Supplement: Supplementary file 1 [file antioxidants-12-01920-s001.zip › antioxidants-2636994-supplementary.pdf]

Supplementary Materials

Table S1. List of primers used in qRT-PCR

Type	Gene symbol	Forward primer (5′–3′)	Reverse primer (5′–3′)	Zebrafish gene ID
Control	***actb1*** (*β-actin*)	GTGATGGTTGGC ATGGGACAG’	CCAGTTGGTCACAAT ACCGTGC	NM_131031.2
Mitochondria genome maintenance	***twnk***	TGTGGGCTGACA AGTTTGAGG	TGTCCACAGACAGAT TTTCTTG	NM_00127759 8.2
	tk2	CCTGTATGAGGA CTGGCTGA	TCTGTTCTCCTCAAAC TGATGC	XM_01735684 5.2
	polg	GGTGACCAGTGA AGACCGATA	GTCCACTGCGCTAAA GAAGG	XM_00192109 5.6
	tfam	GCGAAAGATTGC CCAGCAGT	TTGTCGTTTTTCCTCC GCAAA	NM_00107738 9.1
Mitochondria fission and fusion	***dnm1l***	CGCAGAGTAGCG GGAAGAG	CATCCACTCCGTTCTC CTCG	XM_00516304 9.2
	mfn2	GGCAGGACCAGT AACGGAAA	TTTCGTCCGACCCTTC AGTG	XM_00517248 4.4
Mitochondria respiratory complexes I–V	***ndufs4***	TGTAGGCTGGCA GAGGGACA	GACAGGCCGAAACAG GATGG	NM_00102067 6.1
	sdha	TGGTATGCCGTT CAGCCGTA	GGCCAAGTCTTTGGC ATTGG	NM_200910.1

	uqcrc2b	GACCTCACGGGAAGGGTGAA	TCAGTGTGCTGGTGCTGCTG	NM_001001589.1
	cox5ab	GGTCACCGGAGCTTCAGGAT	TCGAGCCGAGAGGTAGAAAAACC	NM_001305577.1
	atp5fa1	TTCTTGGAGCCGACACTGGA	CGAACACCACAACACCAACG	NM_001077355.1
Oxidative stress	***nfe2l2a***	ATGTCTAAAATGCAGCCAAGCC	CGGTAGCTGAAGTCGAACAC	XM_005172511.4
	sod1	GGGCCAACCGATAGTGTGAG	ACCCTTCCCCAAGTCATCCT	NM_131294.1
	sod2	CCGGACTATGTTAAAGCCATCT	ACACTCGGTTGCTCTCTTTTCTCT	NM_199976.1
	gpx1a	AGATGTCATTCCTGCACACG	AAGGAGAAGCTTCCTCAGCC	NM_001007281.2
Apoptotic marker	***bcl2a***	AGGAAAATGGAGGTTGGGATG	TGTTAGGTATGAAAACGGGTGGA	NM_001030253.2
	baxa	GGCTATTTCAACCAGGGTTCC	TGCGAATCACCAATGCTGT	NM_131562.2
	tp53	GGGCAATCAGCGAGCAAA	ACTGACCTTCCTGAGTCTCCA	XM_005165104.4
	casp9	AAATACAGAGCAAGGCAACC	CACAGGGAATCAAGAAAGG	NM_001007404.2

	apaf1	TTCTACAGTAAACGCCCACC	TATCTAGTATTTCCCCATATTCC	XM_021475139.1
	casp3a	CCGCTGCCCATCACTA	ATCCTTTCACGACCATCT	NM_131877.3
	casp8	GGAATGATCTGGAAGCCTGGG	TCCGGCAAAAGGCAGTGTAA	NM_131510.2
Necroptotic marker	***ripk1l***	TCCTGGACCAAACCATCAGC	ACTGACTCCTCAATTCGGGC	NM_001043350.1
	ripk3	AGCAAAGCCTTCGACGTGTA	CATCCAGTCCTTCGGTCTCG	XM_001343791.5
Heat shock protein marker	***hsp70l***	CGACCTCTTCAGGGGAACACTA	CACCGCTTCATCAGGGTTAATG	NM_001113589.1
Autophagy receptor marker	***sqstm1***	CCCCCTTGGCATAGATGTGG	TTGTTCCCTCACTGACGCTC	NM_001312913.1
Glucose transporter marker	***slc2a1a***	GACCGGCCCATACGTTTTC	ATCATCTCGGTTATATTTATCTGCC	NM_001039808.1
	slc2a2	GCAGAAGAACCCTCACTC	TCTCCGCCACAATAAACC	NM_001042721.1
Neurotransmitter marker	***slc17a6b***	GAACTGACCGAGGATGGACG	TTGCTTTTATGGCCACGCCT	NM_001005398.1
	gad1b	GTTCAGCCATCCTGGTCAGA	CCTTGTCTCCGGTGTCGTAG	XM_009302104.3

	th	GACGGAAGATGATCGGAGACA	CCGCCATGTTCCGATTTCT	NM_131149.1
Oligodendrocytes/ myelin marker	***olig1***	CGGACTGAAAGTTTGAAGAATGC	TCCTGTTACCCGTACCATTCTTG	XM_005167653.4
	olig2	ATCCGTCCAGTTGTGGCACT	TGGTGGAAGCAGAGGATGGT	NM_178100.1
	mbpa	CCGTCGTGGAGACGTCAA	CGAGGAGAGGACACAAAGCT	XM_021468312.1

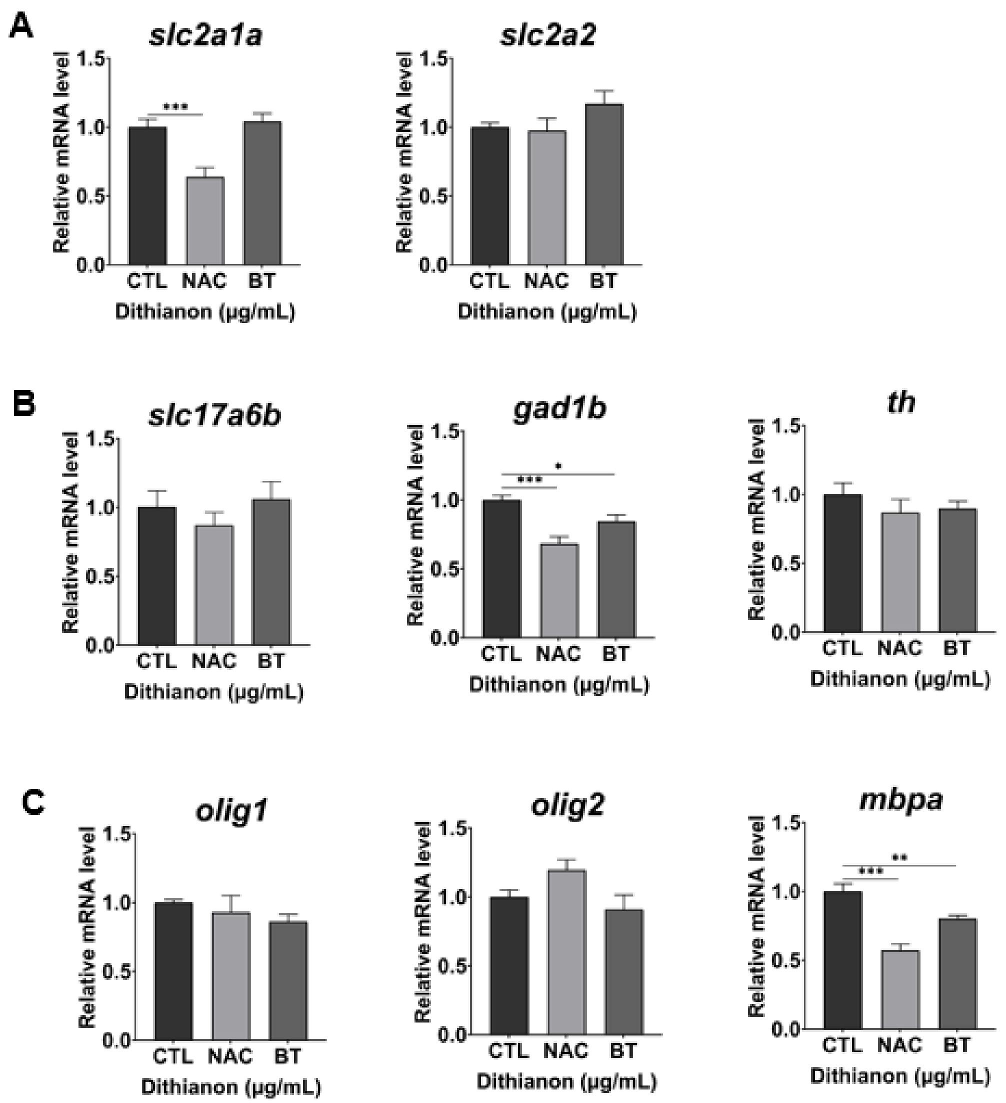

Figure S1. Effects of N-acetylcysteine and betaine in 3 dpf zebrafish larvae.

(**A**) The mRNA expression analysis of the glucose transporters *slc2a1a* and *slc2a2* in 3 dpf larvae treated with 50 μM N-acetylcysteine (NAC) or 50 mM betaine (BT) using qRT-PCR. (**B**) mRNA expression of neuronal genes *slc17a6b*, *gad1b*, and *th*, all of which are involved in major neurotransmission systems. (**C**) mRNA expression of glial genes, *olig1*, *olig2*, and *mbpa*. The mRNA expression data are provided as the means ± SD ($n = 3$). *$p < 0.05$, **$p < 0.01$, ***$p < 0.001$. CTL, control; NAC, N-acetylcysteine; BT, betaine.
